# Impaired renal blood perfusion is closely related to right heart volume overload in congestive heart failure

**DOI:** 10.1186/s43044-025-00692-6

**Published:** 2025-10-10

**Authors:** Ruisi Liu, Yuqing Yang, Lizhuo Li, Qingzhen Zhao, Yuzhi Zhen, Chao Liu, Yue Li

**Affiliations:** 1 Xiangya School of Public Health, Central South University, Hunan, China; 2 The First Hospital of Hebei Medical University, Hebei, China; 3 The Second Hospital of Hebei Medical University, Hebei, China

**Keywords:** Heart failure, Renal circulation, Volume overload

## Abstract

**Background:**

This study investigated the impact of right heart volume overload on renal perfusion in patients with heart failure (HF). We retrospectively analyzed 304 ambulatory HF patients enrolled between October 2017 and August 2022. Echocardiographic parameters—including left atrial diameter (LAD), left ventricular end-diastolic diameter (LVEDD), right atrial diameter (RAD), right ventricular diameter (RVD), and left ventricular ejection fraction (LVEF)—were measured and adjusted for body surface area (BSA). Renal perfusion was assessed via time-to-peak of renal blood flow (TTPr) using renal scintigraphy. Relationships between echocardiographic measures and TTPr were evaluated using Spearman correlation and multivariable ordinal logistic regression analyses.

**Results:**

In the overall cohort, RAD/BSA showed the strongest correlation with TTPr (rs = 0.608, *P* < 0.001), which remained significant after multivariable adjustment. LVEDD/BSA and LVEF showed weak or no associations. Subgroup analyses demonstrated that RAD/BSA had the strongest correlation in HF with reduced ejection fraction (rs = 0.602, *P* < 0.001) and HF with preserved ejection fraction (rs = 0.496, *P* < 0.001), while LAD/BSA was most strongly correlated in HF with mildly reduced ejection fraction (rs = 0.586, *P* < 0.001), all remaining significant after adjustment.

**Conclusions:**

Echocardiographic parameters of the right heart volume overload were associated with TTPr, suggesting a connection between right heart overload and renal perfusion in cardiorenal syndrome. This points to potential therapeutic targets to improve renal perfusion and outcomes in CHF patients.

## Background

Effective management and study of congestive heart failure (CHF) necessitate a comprehensive understanding of the interplay between cardiac-induced volume overload and renal impairment. This dynamic is central to cardiorenal syndrome (CRS), a condition marked by the bidirectional relationship between cardiac and renal dysfunction, where each organ's failure exacerbates the other's condition. A pivotal aspect of this interaction is the impact of volume overload on renal blood flow, which significantly influences the progression and management of heart failure (HF) [1].

In CHF, volume overload—characterized by dilation of the right heart—leads to increased central venous pressure and subsequent renal venous hypertension [2]. This elevated pressure compromises renal blood flow perfusion, resulting in diminished renal function and worse patient outcomes. Right heart catheterization remains the gold standard for hemodynamic assessment in HF, but its invasiveness and technical complexity limit widespread clinical use [3]**.** In contrast, echocardiographic parameters provide a non-invasive and practical alternative for estimating right heart volume overload. Quantifiable measures such as right atrial diameter (RAD) and right ventricular diameter (RVD) are critical in assessing the extent of right heart volume overload in CHF. Additionally, the time-to-peak of renal blood flow (TTPr), assessed via renal scintigraphy, serves as an effective metric for evaluating the influence of cardiac changes on renal hemodynamics. A delayed TTPr indicates impaired renal perfusion [4, 5], a consequence of elevated venous pressure due to right heart overload, thereby highlighting a potentially modifiable risk factor within the cardiorenal axis. These measures directly reflect the heart's overwhelmed capacity to pump blood and the ensuing pressure burden on renal circulation. The understanding of the hemodynamic interplay between the heart and kidneys may guide new therapeutic strategies focused on reducing right heart overload to enhance renal blood flow and improve overall outcomes in CHF patients.

This study systematically examines and quantifies the association between changes in RAD—an indicator of right heart volume overload and the TTPr of renal blood flow in patients with CHF. By combining detailed echocardiographic assessments of RAD with TTPr data derived from ⁹⁹ᵐTc-DTPA renal scintigraphy, we seek to elucidate the mechanisms by which cardiac volume overload affects renal perfusion.

## Methods

### Study population

A retrospective study was carried out for 304 ambulatory HF patients who attended our institute between October 2017 and August 2022, all of whom were clinically stable after receiving standard guideline-directed therapy. The inclusion criteria were as follows: age ≥ 18 years; symptoms and signs of HF such as dyspnea, reduced exercise tolerance, pulmonary rale, and/or edema; meeting one of the following criteria: left ventricular ejection fraction (LVEF) ≤ 40%; and LVEF 41–49% or LVEF ≥ 50% with evidence of left ventricular diastolic dysfunction/raised filling pressures, including elevated plasma B-type natriuretic peptide (BNP). Subjects were further classified based on the LVEF as follows: HF with reduced ejection fraction (HFrEF, LVEF ≤ 40%); HF with mildly reduced ejection fraction (HFmrEF, LVEF of 41–49%); and HF with preserved ejection fraction (HFpEF, LVEF ≥ 50%). Meanwhile, the exclusion criteria included patients with hemodynamic instability, acute pulmonary embolism, advanced malignant tumors, multi-organ failure, severe valvular heart disease, cor pulmonale, and severe primary kidney disease or significant renal dysfunction as characterized by a glomerular filtration rate (GFR) of < 30 mL/min/1.73 m^2^. The study protocol was approved by the Ethics Committee. All participants provided written informed consent.

### Echocardiography

Routine transthoracic echocardiography was performed according to institutional protocol and ASE/EACVI guidelines [6], using an iE33 ultrasound system with an S5-1 transducer (Philips Healthcare, USA). Examinations were performed with patients in the left lateral decubitus position during quiet respiration. Two-dimensional images were acquired from standard parasternal long-axis and apical four-chamber views at end-diastole. Doppler recordings were obtained from the pulmonary veins, mitral valve, left ventricular outflow tract, and aortic valve. The dimensions of each cardiac chamber, including left atrial diameter (LAD), left ventricular end-diastolic diameter (LVEDD), RAD, RVD, and interventricular septum (IVS), and left ventricular posterior wall (LVPW), were adjusted according to the body surface area (BSA) for the accuracy and comparability. LVEF was calculated by Simpson’s biplane method. All measurements were analyzed offline by an experienced imaging cardiologist.

### Renal scintigraphy

The detailed renal scintigraphy procedure was described in the previous study [3]. In brief, a bolus injection of ^99m^Tc-diethylenetriaminepentaacetic acid (^99m^Tc-DTPA) (111–185 MBq) was administered intravenously, followed by a saline flush. Dynamic imaging was initiated concurrently and continued for 21 min by a gamma camera (Infinia, General Electric Healthcare, USA). Data were captured on a 64 × 64 matrix using an online computer processing system (Xeleris 3 Functional Imaging System). The initial phase consisted of 2 s/frame for the first 60 s, followed by 0.5 min/frame for the next 20 min. Regions of interest (ROIs) for the abdominal aorta and kidneys were manually delineated by a blinded technologist, and first-pass time-activity curves were generated [7, 8]. TTPr was defined as the time from injection to the onset and peak enhancement in the renal ROIs. GFR was calculated using Gates' method [9].

### Statistical analysis

Statistical analysis was performed with SPSS 26.0 and R 4.3.1. Quantitative data were presented as either mean ± standard deviation or median with the first and third quartiles, depending on the data distribution. Two-group comparisons were conducted using either the Student’s *t*-test or the Mann–Whitney *U*-test. Multigroup comparisons employed one-way analysis of variance or the Kruskal–Wallis test. Qualitative data were expressed as percentages and assessed using the Chi-square test or Fisher’s exact test. Spearman correlation analysis was performed to evaluate the relationship between echocardiographic parameters and TTPr. Scatter plots and correlation matrix plots were generated to visualize these associations. Multivariable ordinal logistic regression was applied to identify independent determinants of TTPr. A *P*-value < 0.05 was considered statistically significant.

## Results

A total of 304 ambulatory HF patients were included in the study. All participants were clinically stable under standard guideline-directed therapy at enrollment. The predominant etiologies were ischemic cardiomyopathy secondary to coronary artery disease and dilated cardiomyopathy. There were significant differences in TTPr among the HFrEF, HFmrEF, and HFpEF groups, with median values of 37 (27, 49) s, 27 (23, 37) s, and 21 (18, 27) s, respectively (*P* < 0.001). LAD/BSA, LVEDD/BSA, RAD/BSA, RVD/BSA, and LVEF also showed significant differences among the three groups (*P* < 0.001) (Table 1).

**Table 1:** Clinical characteristics of study subjects (*n* = 304)

	Total (*n* = 304)	HFrEF (*n* = 138)	HFmrE*F* (*n* = 56)	HFpEF (*n* = 110)	*P*
Age (y)	59 (48,67)	54 (43.25,64)	59 (49.25,67.25)	63 (54,71)	< 0.001
Sex					< 0.001
Male	211 (69.41%)	102 (73.91%)	49 (87.5%)	60 (54.55%)	
Female	93 (30.59%)	36 (26.09%)	7 (12.5%)	50 (45.45%)	
Height (cm)	170 (163,173)	170 (164.25,173.75)	170 (167.75,175)	165 (160,170)	< 0.001
Weight (kg)	72 (61,80)	71.5 (62,81)	73.5 (65,81.38)	71 (60,80)	0.392
BSA (m^2^)	1.82 ± 0.2	1.83 ± 0.21	1.86 ± 0.18	1.78 ± 0.19	0.025
Systolic blood pressure (mm Hg)	119 (105,132)	113.5 (101,124)	118 (110,134.25)	127 (113.25,135.75)	< 0.001
Diastolic blood pressure (mm Hg)	76 (66.75,86)	75 (66,83.75)	75.5 (63,86.25)	77 (70,87.75)	0.062
Heart rate (bpm)	77 (66,88)	79 (67.25,89)	73.5 (67.75,82)	75.5 (64,86)	0.151
NYHA					< 0.001
I	43 (14.19%)	2 (1.45%)	2 (3.64%)	39 (35.45%)	
II	54 (17.82%)	18 (13.04%)	13 (23.64%)	23 (20.91%)	
III	89 (29.37%)	40 (28.99%)	20 (36.36%)	29 (26.36%)	
IV	117 (38.61%)	78 (56.52%)	20 (36.36%)	19 (17.27%)	
TTPr (s)	27.25 (21,39)	37 (27,49)	27 (23,37)	21 (18,27)	< 0.001
LAD/BSA (mm/m^2^)	23.4 (20.49,26.53)	25.63 (23.33,28.56)	22.08 (19.94,24.4)	20.82 (18.61,24.25)	< 0.001
LVEDD/BSA (mm/m^2^)	32.77 (27.81,37.74)	37.49 (34.46,41.84)	33.03 (29.42,34.82)	27.08 (25.08,29.04)	< 0.001
LVEF (%)	42 (33,60.25)	32 (29,35)	44 (42,47)	64 (58.25,67.75)	< 0.001
RAD/BSA (mm/m^2^)	20.22 (18.35,23.76)	21.83 (19.35,25.67)	19.26 (18.02,21.68)	19.09 (17.23,21.86)	< 0.001
RVD/BSA (mm/m^2^)	19.22 (17.17,22.17)	20.62 (18.25,24.57)	18.72 (17.15,20.82)	18.09 (16.62,20.74)	< 0.001
IVS/BSA (mm/m^2^)	5.5 (4.88,6.29)	5.07 (4.48,5.78)	5.65 (5.08,6.29)	6.03 (5.39,6.71)	< 0.001
LVPW/BSA (mm/m^2^)	5.18 (4.67,5.72)	5.01 (4.23,5.68)	5.07 (4.72,5.52)	5.45 (4.94,5.99)	< 0.001
GFR(mL/min/1.73m^2^)	70 (55.94,86.14)	71.48 (56.91,86.19)	65.88 (51.71,79.84)	73.48 (60.43,87.18)	0.221
B-type natriuretic peptide (pg/mL)	257 (98,664)	501 (219.5,1121.5)	230.5 (96.5,631.5)	106.5 (28.33,240)	< 0.001
Coronary heart disease	149 (49.01%)	43 (31.16%)	30 (53.57%)	76 (69.1%)	< 0.001
Dilated cardiomyopathy	106 (34.87%)	81 (58.70%)	17 (30.36%)	8 (7.27%)	< 0.001
Hypertrophic cardiomyopathy	11 (3.62%)	2 (1.45%)	1 (1.79%)	8 (7.27%)	0.083
Valvular heart disease	19 (6.25%)	5 (3.62%)	4 (7.14%)	10 (9.09%)	< 0.001
Hypertension	141 (46.38%)	50 (36.23%)	28 (50%)	63 (57.27%)	0.002
Hyperlipidemia	56 (18.42%)	23 (16.67%)	9 (16.07%)	24 (21.82%)	0.144
Diabetes	73(24.01%)	35 (25.36%)	16 (28.57%)	22 (20%)	< 0.001
ACE-Is/ARBs/ARNIs	164 (53.95%)	85 (61.59%)	31 (55.36%)	48 (43.64%)	0.018
Beta-blockers	256 (84.21%)	125 (90.58%)	46 (82.14%)	85 (77.27%)	0.015
Mineralocorticoid receptor antagonists	241 (79.28%)	123 (89.13%)	49 (87.5%)	69 (62.73%)	< 0.001
Diuretics	248 (81.58%)	127 (92.03%)	48 (85.71%)	73 (66.36%)	< 0.001
Digoxin	91 (29.93%)	62 (44.93%)	19 (33.93%)	10 (9.09%)	< 0.001

*ACE-Is* Angiotensin-converting enzyme inhibitors, *ARBs* Angiotensin II receptor blockers, *ARNIs* Angiotensin receptor–neprilysin inhibitors, *BSA* Body surface area, *GFR* Glomerular filtration rate, *HFmrEF* Heart failure with mildly reduced ejection fraction, *HFpEF* Heart failure with preserved ejection fraction, *HFrEF* Heart failure with reduced ejection fraction, *IVS* Interventricular septum, *LAD* Left atrium diameter, *LVEDD* Left ventricular end-diastole diameter, *LVEF* Left ventricular ejection fraction, *LVPW* Left ventricular posterior wall, *NYHA* New York Heart Association, *RAD* Right atrial diameter, *RVD* Right ventricular diameter, and *TTPr* Time-to-peak of renal blood flow

### Correlation analysis between echocardiographic parameters and TTPr

**Fig. 1:**
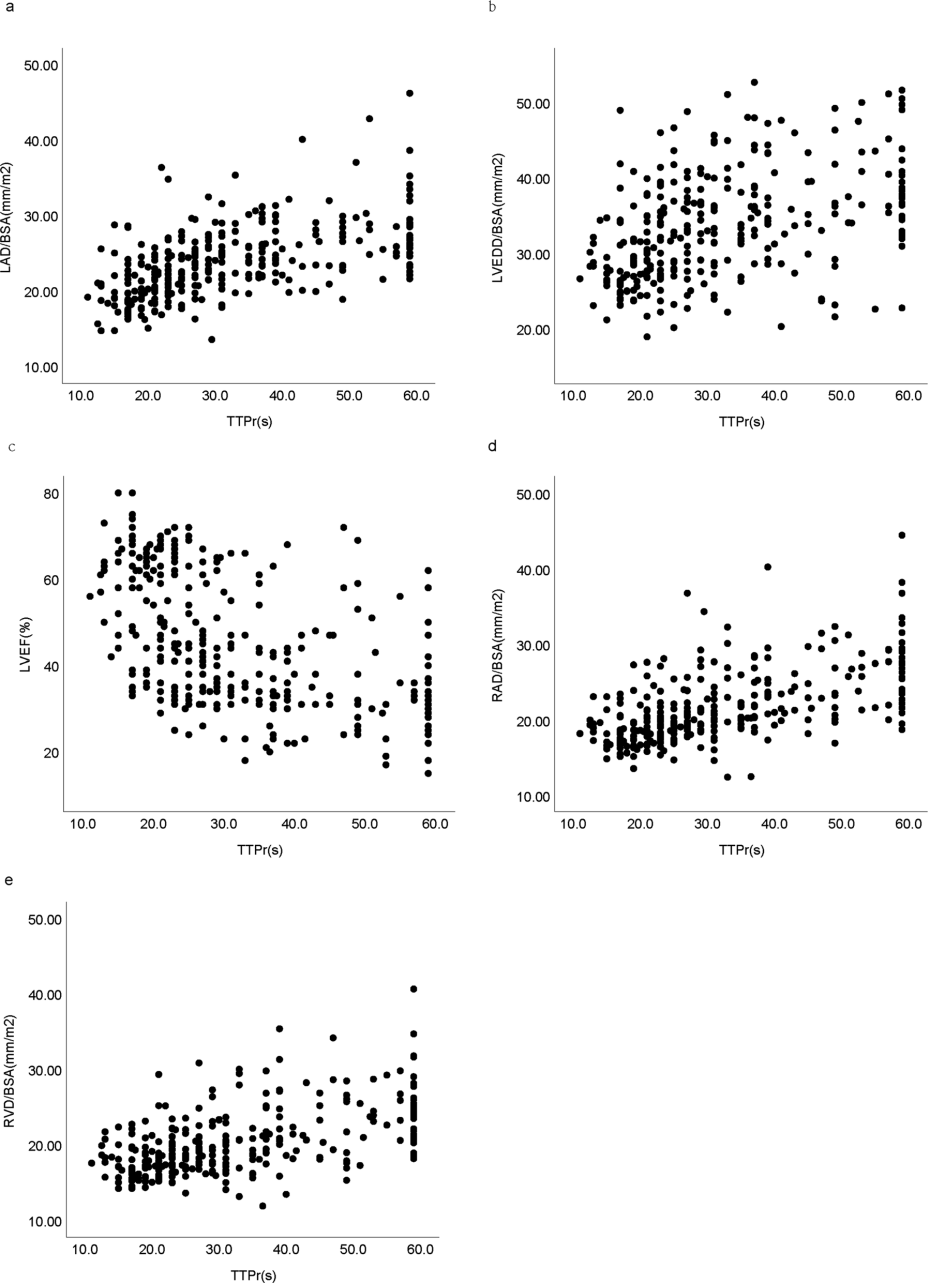
Scatter plots of correlation between TTPr and echocardiographic parameters in HF patients. **a** Scatter plots of correlation between TTPr and LAD/BSA in HF patients. **b** Scatter plots of correlation between TTPr and LVEDD/BSA in HF patients. **c** Scatter plots of correlation between TTPr and LVEF in HF patients. **d** Scatter plots of correlation between TTPr and RAD/BSA in HF patients. **e** Scatter plots of correlation between TTPr and RVD/BSA in HF patients

Spearman correlation analysis was conducted to examine the relationship between echocardiographic parameters and TTPr in HF patients (Table 2), and scatter plots were generated (Fig. 1). RAD/BSA, LAD/BSA, LVEDD/BSA, and RVD/BSA showed positive correlations with TTPr (*P* < 0.001), while LVEF was negatively correlated (*P* < 0.001). After adjustment for relevant covariates (age, height, weight, systolic blood pressure, diastolic blood pressure, heart rate, LAD/BSA, LVEDD/BSA, LVEF, RAD/BSA, RVD/BSA, IVS/BSA, and LVPW/BSA), RAD/BSA, LAD/BSA, and LVEF were independently associated with TTPr (*P* < 0.05) (Table 3). Among these parameters, RAD/BSA had the highest correlation with TTPr (*r*_s_ = 0.608).

**Table 2:** Spearman correlation analysis of echocardiographic parameters and TTPr in heart failure subgroups

	LAD/BSA	LVEDD/BSA	LVEF	RAD/BSA	RVD/BSA	IVS/BSA	LVPW/BSA
TTPr in HF	0.596 (*P* < 0.001)	0.468 (*P* < 0.001)	−.557 (*P* < 0.001)	0.608 (*P* < 0.001)	0.535 (*P* < 0.001)	− 0.16 (*P* = 0.005)	0.009 (*P* = 0.88)
TTPr in HFrEF	0.367 (*P* < 0.001)	0.107 (*P* = 0.213)	− 0.369 (*P* < 0.001)	0.602 (*P* < 0.001)	0.54 (*P* < 0.001)	− 0.025 (*P* = 0.771)	0.026 (*P* = 0.763)
TTPr in HFmrEF	0.586 (*P* < 0.001)	0.177 (*P* = 0.193)	− 0.154 (*P* = 0.258)	0.563 (*P* < 0.001)	0.497 (*P* < 0.001)	0.014 (*P* = 0.919)	0.22 (*P* = 0.104)
TTPr in HFpEF	0.461 (*P* < 0.001)	0.094 (*P* = 0.327)	− 0.19 (*P* = 0.047)	0.496 (*P* < 0.001)	0.404 (*P* < 0.001)	0.212 (*P* = 0.027)	0.347 (*P* < 0.001)

*BSA* Body surface area, *HF* Heart failure, *HFmrEF* Heart failure with mildly reduced ejection fraction, *HFpEF* Heart failure with preserved ejection fraction, *HFrEF* Heart failure with reduced ejection fraction, *IVS* Interventricular septum, *LAD* Left atrium diameter, *LVEDD* Left ventricular end-diastole diameter, *LVEF* Left ventricular ejection fraction, *LVPW* Left ventricular posterior wall, *RAD* Right atrial diameter, *RVD* Right ventricular diameter, and *TTPr* Time-to-peak of renal blood flow

**Table 3:** Multivariable ordinal logistic regression analysis of factors affecting TTPr in heart failure subgroups

	HR	*P*
*HF*		
Age	1.059 (1.037, 1.081)	< 0.001
Height	1.041 (1.003, 1.079)	0.032
Weight	1.042 (1.019, 1.066)	< 0.001
Systolic blood pressure	0.969 (0.952, 0.986)	< 0.001
Diastolic blood pressure	1.048 (1.023, 1.074)	< 0.001
Heart rate	1.006 (0.991, 1.021)	0.441
LAD/BSA	1.132 (1.046, 1.225)	0.002
LVEDD/BSA	0.988 (0.930, 1.050)	0.704
LVEF	0.937 (0.913, 0.962)	< 0.001
RAD/BSA	1.290 (1.158, 1.439)	< 0.001
RVD/BSA	1.057 (0.939, 1.190)	0.361
IVS/BSA	0.874 (0.708, 1.077)	0.205
LVPW/BSA	1.423 (1.041, 1.944)	0.027
*HFrEF*		
Age	1.049 (1.012, 1.088)	< 0.001
Height	1.050 (0.978, 1.128)	0.179
Weight	1.041 (0.998, 1.085)	0.06
Systolic blood pressure	0.969 (0.942, 0.998)	0.034
Diastolic blood pressure	1.041 (1.004, 1.080)	0.031
Heart rate	0.995 (0.970, 1.021)	0.7
LAD/BSA	1.126 (0.980, 1.294)	0.094
LVEDD/BSA	0.974 (0.896, 1.059)	0.54
LVEF	0.885 (0.814, 0.962)	< 0.001
RAD/BSA	1.430 (1.176, 1.742)	< 0.001
RVD/BSA	0.963 (0.799, 1.159)	0.687
IVS/BSA	0.691 (0.481, 0.994)	0.046
LVPW/BSA	1.355 (0.893, 2.054)	0.153
*HFmrEF*		
Age	1.038 (0.977, 1.103)	0.225
Height	1.042 (0.951, 1.140)	0.378
Weight	1.048 (1.002, 1.097)	0.043
Systolic blood pressure	0.992 (0.946, 1.041)	0.742
Diastolic blood pressure	0.990 (0.940, 1.044)	0.717
Heart rate	1.013 (0.975, 1.051)	0.5
LAD/BSA	1.328 (1.043, 1.690)	0.021
LVEDD/BSA	0.982 (0.815, 1.183)	0.849
LVEF	0.886 (0.694, 1.131)	0.331
RAD/BSA	1.129 (0.862, 1.478)	0.377
RVD/BSA	1.132 (0.804, 1.592)	0.478
IVS/BSA	1.102 (0.628, 1.935)	0.736
LVPW/BSA	1.496 (0.652, 3.435)	0.342
*HFpEF*		
Age	1.091 (1.044, 1.140)	< 0.001
Height	1.040 (0.972, 1.113)	0.25
Weight	1.056 (1.000, 1.115)	0.051
Systolic blood pressure	0.938 (0.905, 0.972)	0.001
Diastolic blood pressure	1.162 (1.093, 1.235)	< 0.001
Heart rate	1.006 (0.977, 1.036)	0.671
LAD/BSA	1.111 (0.965, 1.279)	0.144
LVEDD/BSA	1.009 (0.849, 1.200)	0.917
LVEF	0.952 (0.878, 1.033)	0.239
RAD/BSA	1.519 (1.231, 1.874)	< 0.001
RVD/BSA	1.060 (0.839, 1.338)	0.626
IVS/BSA	0.920 (0.644, 1.315)	0.648
LVPW/BSA	2.433 (1.074, 5.510)	0.033

*BSA* Body surface area, *HF* Heart failure, *HFmrEF* Heart failure with mildly reduced ejection fraction, *HFpEF* Heart failure with preserved ejection fraction, *HFrEF* Heart failure with reduced ejection fraction, *IVS* Interventricular septum, *LAD* Left atrium diameter, *LVEDD* Left ventricular end-diastole diameter, *LVEF* Left ventricular ejection fraction, *LVPW* Left ventricular posterior wall, *RAD* Right atrial diameter, *RVD* Right ventricular diameter, and *TTPr* Time-to-peak of renal blood flow

### Correlation analysis of echocardiographic parameters and TTPr in different subgroups of HF

**Fig. 2:**
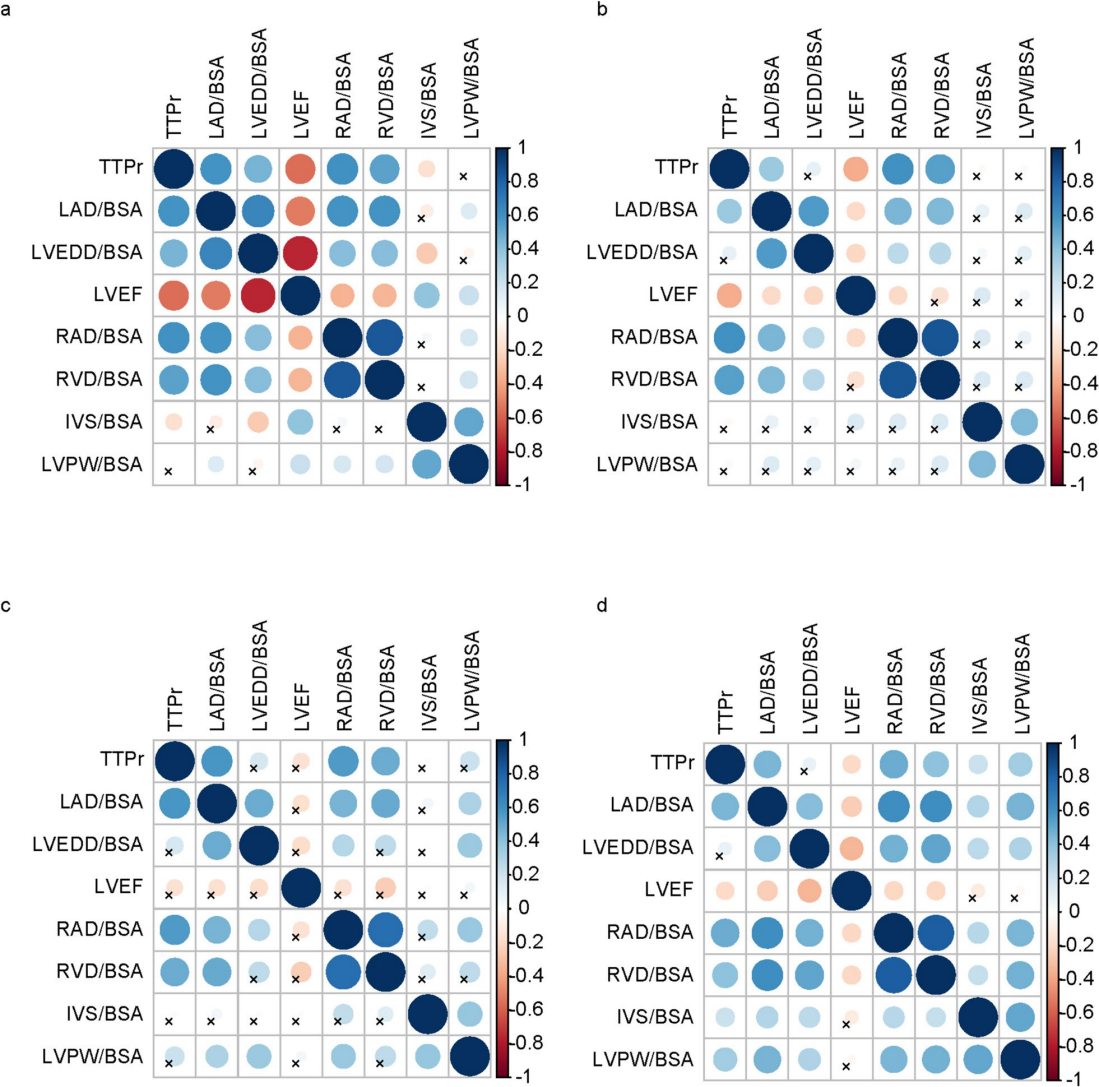
The correlation matrix of echocardiographic parameters and TTPr. **a** The correlation matrix of echocardiographic parameters and TTPr in total HF patients. **b** The correlation matrix of echocardiographic parameters and TTPr in HFrEF group. **c** The correlation matrix of echocardiographic parameters and TTPr in HFmrEF group. **d** The correlation matrix of echocardiographic parameters and TTPr in HFpEF group

Spearman correlation analysis revealed that RAD/BSA exhibited the strongest correlation with TTPr in the HFrEF and HFpEF groups (*r*_s_ = 0.602 and 0.496), followed by RVD/BSA and LAD/BSA (*P* < 0.001). After adjustment for relevant covariates, only RAD/BSA was independently associated with TTPr (*P* < 0.05). In the HFmrEF group, LAD/BSA showed the strongest correlation with TTPr (*r*_s_ = 0.586), followed by RAD/BSA and RVD/BSA (*P* < 0.001). After adjustment for relevant covariates, only LAD/BSA was independently associated with TTPr (*P* < 0.05). In all subgroups, LVEDD/BSA and LVEF demonstrated weak or no correlation (Tables 2 and 3, Fig. 2).

### Correlation analysis of TTPr with other classic congestion markers

**Fig. 3:**
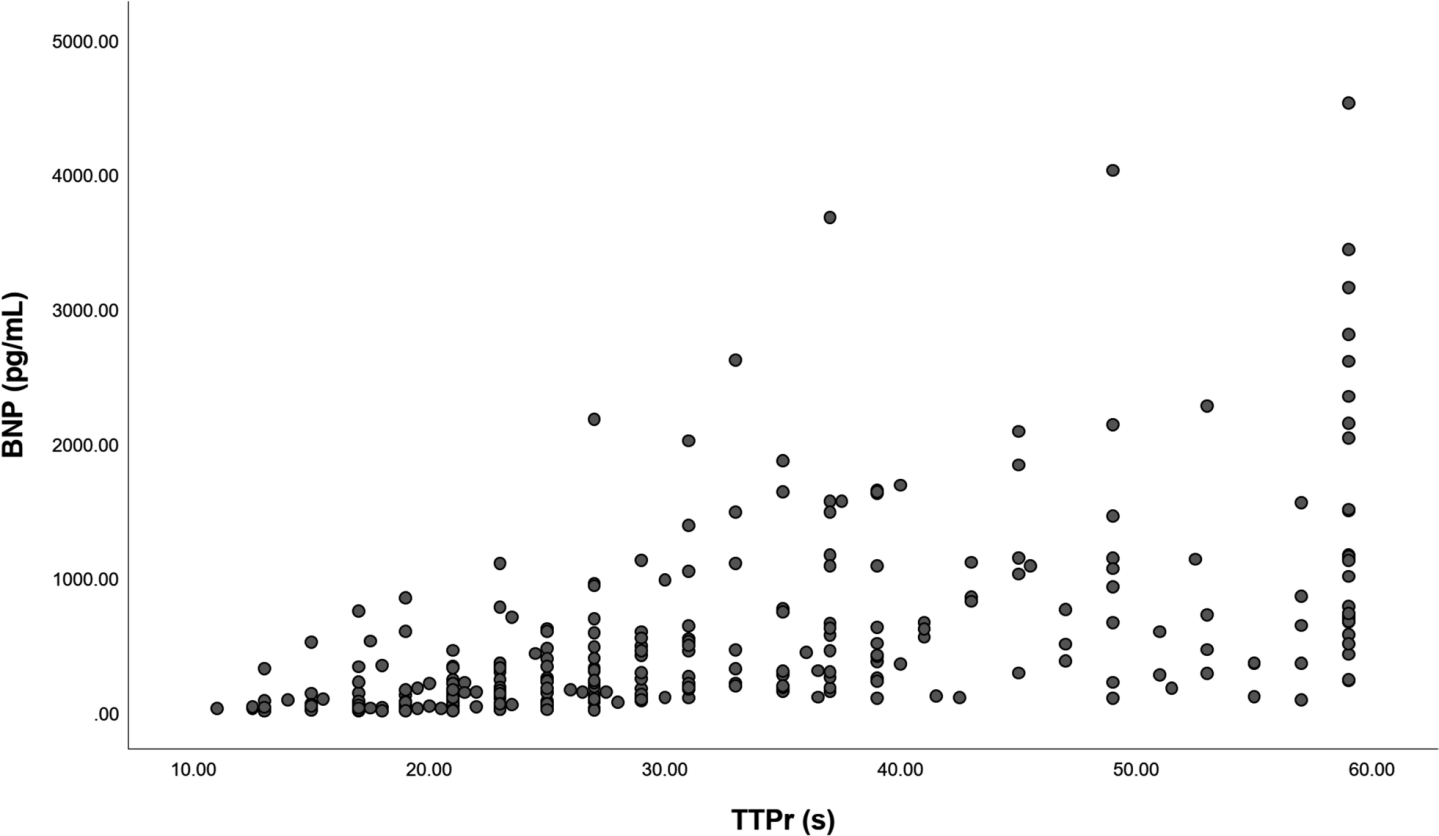
Scatter plots of correlation between TTPr and BNP

Spearman correlation analysis revealed that TTPr was significantly positively correlated with BNP (*r*s = 0.695, *P* < 0.001) (Fig. 3).

## Discussion

Our study demonstrates a significant relationship between RAD and TTPr, indicating an association between right heart volume overload and impaired renal perfusion. The study cohort mainly included patients with ischemic cardiomyopathy due to coronary artery disease and dilated cardiomyopathy. Importantly, patients with significant primary valve disorders or other conditions (e.g., cor pulmonale) that could independently cause right heart dilatation were excluded. Nearly half had hypertension and about one-fourth had diabetes. All were clinically stable under guideline-directed therapy. Patients with severe renal dysfunction or chronic kidney disease were excluded to reduce confounding, allowing our findings to more specifically reflect the impact of cardiac dysfunction on renal perfusion and CRS mechanisms.

RAD and RVD are indicators of right heart volume overload, and their elevation directly correlates with right atrial and ventricular remodeling under chronic volume overload [10]. Compared with the right ventricle, the right atrium tends to dilate earlier and more prominently due to its thinner wall, higher compliance, and greater sensitivity to venous pressure elevation, making RAD a particularly sensitive marker of right heart volume overload [11–13].

In both HFrEF and HFpEF, RAD was observed to be the parameter most strongly correlated with TTPr, suggesting that right heart volume overload adversely affects renal hemodynamics [10]. This relationship indicates the importance of managing volume overload to mitigate its detrimental effects on renal function, consistent with existing literature on the pathophysiology of congestion and renal venous hypertension [14, 15]. However, in HFmrEF, with relatively preserved systolic and diastolic function, left ventricular filling pressures are lower, and right heart volume overload is less pronounced. Consequently, right heart parameters are less sensitive, while the left atrium, adjacent to the left ventricle, may more directly reflect hemodynamic alterations, explaining its stronger correlation with TTPr in this subgroup [16–18].

Across all HF subgroups, TTPr showed a weak or insignificant correlation with LVEF, particularly in HFrEF. Simultaneously, TTPr demonstrated a significant positive correlation with the classical congestion marker BNP. These findings suggest that renal perfusion is more closely related to right heart volume overload, with renal venous congestion rather than reduced LVEF being the main driver of CRS [1, 19, 20]. However, it should be recognized that increased cardiac volume overload is not the sole cause of impaired renal perfusion, neurohormonal activation and systemic hemodynamic changes may play an equal or even greater role [21]. Incorporating additional markers, such as the hemoglobin-to-creatinine ratio [22], may offer complementary and easily measurable tools for elucidating CRS mechanisms and guiding monitoring or intervention.

The previous studies used Doppler ultrasound to assess renal venous flow in acute HF and explored right heart pulmonary circulation coupling, a method gaining traction for its practicality and low cost [23]. In contrast, we chose renal scintigraphy because it is highly feasible and does not require prolonged breath-holding. More importantly, it allows accurate measurement of GFR, which is essential for CRS research.

The findings of our study suggest that therapeutic strategies aimed at reducing right heart volume overload may potentially improve renal perfusion. Such strategies could include optimized fluid management, the use of diuretics, and possibly interventions to improve right ventricular function [24, 25]. Furthermore, renal scintigraphy could serve as a non-invasive tool to identify hospitalized HF patients at higher risk of renal hypoperfusion, allowing for earlier and more tailored optimization of therapy. Integrating renal perfusion assessment into routine evaluation may help clinicians adjust decongestive strategies more precisely, avoid excessive diuresis, and potentially reduce the risk of renal dysfunction and HF rehospitalization. Regular monitoring of RAD and TTPr may, therefore, be crucial in adjusting treatment plans for patients with CHF, thus providing a targeted approach to the management of CRS.

## Limitations

This study has several important limitations. First, its retrospective, single-center, cross-sectional design may introduce selection bias, restrict external validity, and limit causal inference. Second, only structural echocardiographic variables were included, without right heart functional or hemodynamic parameters (e.g., TAPSE, RV FAC, etc.), which may confound the observed associations. Third, the absence of longitudinal follow-up prevents assessment of long-term outcomes and the prognostic significance of TTPr. Fourth, the relatively small sample size, particularly in some subgroups, may limit the robustness of findings. Fifth, all participants were clinically stable HF patients, which may restrict generalizability to more severe or decompensated HF. Finally, residual confounding cannot be fully excluded. Future prospective, multicenter, longitudinal, and interventional studies are needed to validate these findings and clarify their clinical implications.

## Conclusion

In conclusion, our study suggests that right heart volume overload has a significant impact on renal perfusion, highlighting a potential pathophysiological link within CRS. These findings add to our understanding of the mechanisms underlying CRS and could indicate potential therapeutic targets to improve clinical outcomes in patients with CHF.

## Data Availability

The datasets used and analyzed during the current study are available from the corresponding author upon request.

## Abbreviations

CHF: Congestive heart failure
CRS: Cardiorenal syndrome
HF: Heart failure
RAD: Right atrial diameter
RVD: Right ventricular diameter
TTPr: Time-to-peak of renal blood flow
LVEF: Left ventricular ejection fraction
BNP: B-type natriuretic peptide
HFrEF: Heart failure with reduced ejection fraction
HFmrEF: Heart failure with mildly reduced ejection fraction
HFpEF: Heart failure with preserved ejection fraction
GFR: Glomerular filtration rate
LAD: Left atrial diameter
LVEDD: Left ventricular end-diastolic diameter
BSA: Body surface area

## References

[1] Ronco C, McCullough PA, Anker SD et al (2010) Cardiorenal syndromes: an executive summary from the consensus conference of the Acute Dialysis Quality Initiative (ADQI). Contrib Nephrol 165:54–67 20427956 10.1159/000313745

[2] Chen Y, He XM, Meng H et al (2014) Relationship between lipids levels and right ventricular volume overload in congestive heart failure. J Geriatr Cardiol 11:192–199 25278966 10.11909/j.issn.1671-5411.2014.03.011 PMC4178509

[3] Manzi L, Sperandeo L, Forzano I et al (2024) Contemporary evidence and practice on right heart catheterization in patients with acute or chronic heart failure. Diagnostics 14:136 38248013 10.3390/diagnostics14020136 PMC10814482

[4] Ma H, Gao X, Yin P et al (2021) Semi-quantification of renal perfusion using (99m)Tc-DTPA in systolic heart failure: a feasibility study. Ann Nucl Med 35:187–194 33386522 10.1007/s12149-020-01556-6

[5] Komuro K, Seo Y, Yamamoto M et al (2018) Assessment of renal perfusion impairment in a rat model of acute renal congestion using contrast-enhanced ultrasonography. Heart Vessels 33:434–440 29027577 10.1007/s00380-017-1063-7

[6] Lang RM, Badano LP, Mor-Avi V (2015) Recommendations for cardiac chamber quantification by echocardiography in adults: an update from the American Society of Echocardiography and the European Association of Cardiovascular Imaging. J Am Soc Echocardiogr 28:1-39.e14 25559473 10.1016/j.echo.2014.10.003

[7] O’Reilly PH (2003) Standardization of the renogram technique for investigating the dilated upper urinary tract and assessing the results of surgery. BJU Int 91:239–243 12581012 10.1046/j.1464-410x.2003.04050.x

[8] Taylor AT, Blaufox MD, De Palma D et al (2012) Guidance document for structured reporting of diuresis renography. Semin Nucl Med 42:41–48 22117812 10.1053/j.semnuclmed.2010.12.006 PMC3226810

[9] Narayan ML, Jain S, Ashok K, Dhingra VK (2018) Comparison of glomerular filtration rate calculated by Gamma camera-based Gates method with various estimated glomerular filtration rate methods

[10] Thandavarayan RA, Chitturi KR, Guha A (2020) Pathophysiology of acute and chronic right heart failure. Cardiol Clin 38:149–160 32284093 10.1016/j.ccl.2020.01.009 PMC11976311

[11] Reddy S, Zhao M, Hu DQ et al (2013) Physiologic and molecular characterization of a murine model of right ventricular volume overload. Am J Physiol Heart Circ Physiol 304:H1314-1327 23504182 10.1152/ajpheart.00776.2012 PMC3652064

[12] Havlenova T, Skaroupkova P, Miklovic M et al (2021) Right versus left ventricular remodeling in heart failure due to chronic volume overload. Sci Rep 11:17136 34429479 10.1038/s41598-021-96618-8 PMC8384875

[13] Chemla D, Berthelot E, Assayag P et al (2018) Physiopathologie hémodynamique du ventricule droit. Rev Mal Respir 35:1050–1062 29945812 10.1016/j.rmr.2017.10.667

[14] Mori T (2024) Renal venous hypertension to the regulation of pressure natriuresis in heart failure. Hypertens Res 47:1081–1083 38228752 10.1038/s41440-023-01512-7

[15] Miller WL (2016) Fluid volume overload and congestion in heart failure: time to reconsider pathophysiology and how volume is assessed. Circ Heart Fail 9:e002922 27436837 10.1161/CIRCHEARTFAILURE.115.002922

[16] Triposkiadis F, Pieske B, Butler J et al (2016) Global left atrial failure in heart failure. Eur J Heart Fail 18:1307–1320 27813305 10.1002/ejhf.645

[17] Ltaief Z, Yerly P, Liaudet L (2023) Pulmonary hypertension in left heart diseases: pathophysiology, hemodynamic assessment and therapeutic management. Int J Mol Sci 24:9971 37373119 10.3390/ijms24129971 PMC10298585

[18] van Wezenbeek J, Kianzad A, van de Bovenkamp A et al (2022) Right ventricular and right atrial function are less compromised in pulmonary hypertension secondary to heart failure with preserved ejection fraction: a comparison with pulmonary arterial hypertension with similar pressure overload. Circ Heart Fail 15:e008726 34937392 10.1161/CIRCHEARTFAILURE.121.008726 PMC8843396

[19] Husain-Syed F, Gröne HJ, Assmus B et al (2021) Congestive nephropathy: a neglected entity? Proposal for diagnostic criteria and future perspectives. ESC Heart Fail 8:183–203 33258308 10.1002/ehf2.13118 PMC7835563

[20] Grande D, Terlizzese P, Iacoviello M (2017) Role of imaging in the evaluation of renal dysfunction in heart failure patients. World J Nephrol 6:123–131 28540202 10.5527/wjn.v6.i3.123 PMC5424434

[21] Kim JA, Wu L, Rodriguez M (2023) Recent developments in the evaluation and management of cardiorenal syndrome: a comprehensive review. Curr Probl Cardiol 48:101509 36402213 10.1016/j.cpcardiol.2022.101509

[22] Ikuta A, Oka S, Matsushita S et al (2023) Impact of serum haemoglobin-to-creatinine ratio after transcatheter aortic valve implantation. Open Heart 10:e002419 38042526 10.1136/openhrt-2023-002419 PMC10693869

[23] Vella A, Labate V, Carenini G (2023) Phenotyping congestion in acute heart failure by renal flow and right heart to pulmonary circulation coupling. ESC Heart Fail 10:3546–3558 37743691 10.1002/ehf2.14522 PMC10682856

[24] Houston BA, Kalathiya RJ, Kim DA, Zakaria S (2015) Volume overload in heart failure: an evidence-based review of strategies for treatment and prevention. Mayo Clin Proc 90:1247–1261 26189443 10.1016/j.mayocp.2015.05.002

[25] Stickel S, Gin-Sing W, Wagenaar M, Gibbs J (2019) The practical management of fluid retention in adults with right heart failure due to pulmonary arterial hypertension. Eur Heart J Suppl 21:K46–K53 31857800 10.1093/eurheartj/suz207 PMC6915055

